# HPMPdb: A machine learning-ready database of protein molecular phenotypes associated to human missense variants

**DOI:** 10.1016/j.crstbi.2022.04.004

**Published:** 2022-05-13

**Authors:** Daniele Raimondi, Francesco Codicè, Gabriele Orlando, Joost Schymkowitz, Frederic Rousseau, Yves Moreau

**Affiliations:** a ESAT-STADIUS, KU Leuven, Kasteelpark Arenberg 10, Leuven, 3001, Belgium; b Department of Pharmacy and Biotechnology, Via San Giacomo 9/2, 40126, Bologna, Italy; c Switch Laboratory, VIB-KU Leuven Center for Brain and Disease Research, Herestraat 49, 3000 Leuven, Belgium; 4 Switch Laboratory, Department of Cellular and Molecular Medicine, KU Leuven, Herestraat 49, 3000 Leuven, Belgium

**Keywords:** Single aminoacid variants, Molecular phenotype, Variant-effect predictor, Database, Bioinformatics

## Abstract

Current human Single Amino acid Variants (SAVs) databases provide a link between a SAVs and their effect on the carrier individual phenotype, often dividing them into Deleterious/Neutral variants. This is a very coarse-grained description of the genotype-to-phenotype relationship because it relies on un-realistic assumptions such as the perfect Mendelian behavior of each SAV and considers only dichotomic phenotypes. Moreover, the link between the effect of a SAV on a protein (its molecular phenotype) and the individual phenotype is often very complex, because multiple level of biological abstraction connect the protein and individual level phenotypes. Here we present HPMPdb, a manually curated database containing human SAVs associated with the detailed description of the molecular phenotype they cause on the affected proteins. With particular regards to machine learning (ML), this database can be used to let researchers go beyond the existing Deleterious/Neutral prediction paradigm, allowing them to build molecular phenotype predictors instead. Our class labels describe in a succinct way the effects that each SAV has on 15 protein molecular phenotypes, such as protein-protein interaction, small molecules binding, function, post-translational modifications (PTMs), sub-cellular localization, mimetic PTM, folding and protein expression. Moreover, we provide researchers with all necessary means to re-producibly train and test their models on our database. The webserver and the data described in this paper are available at hpmp.esat.kuleuven.be.

## Highlights

• Current variant-effect predictors perform a coarse-grained modeling and rely on unrealistic assumptions.
• The link between the effect of a variant and the individual phenotype is complex. It would be more intuitive to predict the molecular phenotype that each variant causes on the carrier protein.
• HPMP is a manually curated database containing human variants associated with the molecular phenotype they cause on the affected proteins.
• We manually translated variants from Uniprot into 15 Machine Learning-ready labels describing the affected protein molecular phenotype.
• The goal of HPMP is to allow researchers to go beyond the existing variant-effect prediction paradigm and allow them to build molecular phenotype predictors instead.
• The webserver and the data described in this paper are available at hpmp.esat.kuleuven.be

## Graphical abstract

**Figure:**
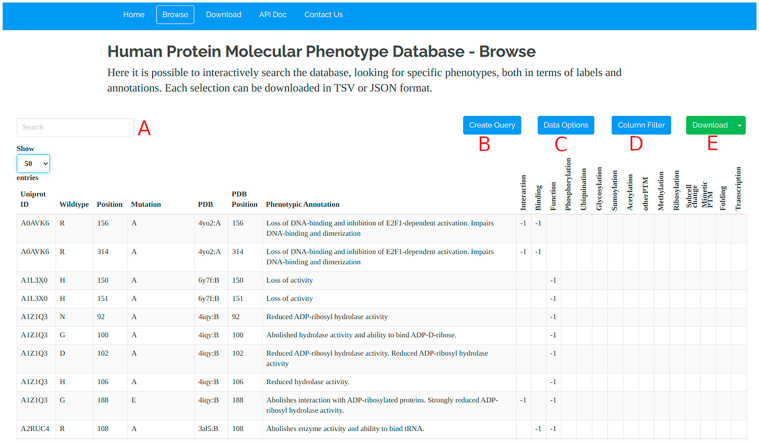

## 1 Introduction

Understanding the relationship between genotype and phenotype is one of the most relevant goals of Biology and Bioinformatics, and in the last decades, a lot of efforts have been put into developing Bioinformatics tools able to predict the effect of Single Amino acid Variants (SAVs) in Humans. Several variant-effect predictors (also called pathogenicity predictors) have been published (e.g. (Adzhubei et al., 2010; Ng and Henikoff, 2001; Shihab et al., 2013; Raimondi et al., 2016; Raimondi et al., 2017; Dong Peng et al., 2015; Jagadeesh et al., 2016)) finding also application in genomic pipelines (Pejaver et al., 2020).

These tools can be roughly divided into two main categories. The largest set contains Machine Learning (ML)-based methods that generally aggregate various heterogeneous information describing the SAV and the protein on which it occurs and feed it as features into ML models such as Random Forests, Support Vector Machines and Neural Networks (Raimondi et al., 2016; Raimondi et al., 2017; Dong Peng et al., 2015, Jagadeesh et al., 2016; Adzhubei et al., 2010; Dong Peng et al., 2015). The second category contains the relatively few empirical models, based on a priori statistical modeling of the perceived impact that the target mutations has on the protein from an evolutionary perspective (Ng and Henikoff, 2001; Choi et al., 2012).

Regardless of the category, all variant-effect predictors take as input the protein-level residue change produced by a single DNA-level missense variants and try to predict its effect for the carrier organism, benchmarking and/or training on datasets such as HGMD (Stenson et al., 2009), Humsavar (Apweiler et al., 2004), ClinVar (Landrum et al., 2016). The main characteristic of these datasets is that they associate SAVs with individual-level phenotypes, and thus, by training and testing on them, the learned models implicitly adopt strong assumptions, such as the perfect Mendelian genetic behavior of each SAV.

Moreover, the vast majority of these tools only aim at the generic distinction between the “deleterious” and “neutral” individual-level phenotypic effects, without trying a more detailed phenotypic characterization (Pejaver et al., 2020). The recently published MutPred2, which indeed predicts also the SAVs' protein molecular phenotype, is the only exception to this trend that we are aware of (Pejaver et al., 2020). It uses a positive-unlabeled approach to the prediction of 50 molecular phenotypes, highlighting that i) this kind of training data is currently lacking and that ii) the Bioinformatics community is indeed ready to at least start complementing the classical Deleterious/Neutral variant-effect prediction paradigm with molecular phenotype predictions.

The real connection between a SAV and the phenotype of the carrier individual is, in the vast majority of the cases, much more complex (Pejaver et al., 2020). Each SAV is directly only responsible for a protein-level change (e.g. a protein molecular phenotype alteration), which is then modulated up to the individual phenotype through the complex networks of feedback and interactions that regulate cell life, tissue development and the general organism's welfare. Associating binary individual-level phenotypes to SAVs is thus a harsh simplification that was nonetheless a necessary step to allow the initial the development of variant effect predictors, due to the lack of more detailed data regarding the molecular-level effect of SAVs.

To overcome these conceptual limitations, and to allow the development of more specific variant-effect predictors, able to identify the protein molecular phenotype directly caused by each SAV, in this paper we propose a novel, manually-curated, ML-ready Human Protein Molecular Phenotype database (HPMPdb). HPMPdb contains more than 8000 SAVs annotated with the molecular phenotype they cause on the proteins on which they are mapped. To collect this data we mined all the SAVs annotated with a molecular phenotype description in Unipro-tKB (Apweiler et al., 2004) and we manually translated this description to one or more of 15 specific molecular phenotype classes, that can be directly used as ground truth labels to train ML models.

These classes indicate whether a SAV affects the protein function, its interactions, its binding affinity, its subcellular localization, its post-translational modifications (PTMs) or expression and they can be natively used as labels for the development of future variant-effect predictors that will directly aim at the multi-class or multi-label prediction of the molecular phenotypes associated to each SAVs, thus relaxing the strong assumptions adopted by the existing variant-effect prediction paradigm, which aims at the binary discrimination between “deleterious” and “neutral” SAVs. The database is freely available at hpmp.esat.kuleuven.be.

## 2 Methods

### 2.1 Single Amino acid Variants collection and annotation

We mined UniprotKB (Apweiler et al., 2004) collecting all the Human SAVs mentioned in the “Disruption phenotype”, “Mutagenesis” and “Involvement in disease” fields, obtaining 7235 proteins. To ensure a higher degree of reliability of the SAVs, we then used SIFTS (Velankar et al., 2012) to map these SAVs on PDB (Berman et al., 2000) protein structures, finally obtaining 8007 SAVs mapped on 1766 proteins.

Each SAV is also annotated on Uniprot with a short natural language description of the phenotypic effect it produces on the target protein. These descriptions have been manually extracted from the corresponding publication(s) in which the phenotype caused by the SAV has been experimentally determined. Detailed examples of these descriptions are provided in the following sections. All phenotypic annotations in HPMPdb are therefore derived from at least one published paper. As a next step, we manually translated these molecular phenotype effect annotations for each SAV into the following 15 molecular phenotype classes: interaction, binding, function, post-translational modifications (PTMs) (e.g. phosphorylation, ubiquination, glycosylation, sumoylation, acetylation, methylation, ribosylation, other PTM), subcellular lo-calization change, mimetic PTM, folding and expression. A detailed description of each class is provided in the next section.

### 2.2 Criteria for the definition of the molecular phenotype classes

We now describe in detail the criteria used to determine each molecular phenotypic class. In the following, we will refer to proteins using their Uniprot ID (e.g. Q8K9I1) and to SAVs as wildtype residue, position and mutant residue, using the amino acids single letter code (e.g. A234K indicates that the wildtype Alanine at position 234 is mutated into a Lysine).

#### 2.2.1 Interaction

This molecular phenotype class describes the effects that a SAV has on the affected protein's interactions with other proteins (but *not* small molecules and nucleic acids). In particular we used the label −1 if the SAV negatively affects the interaction(s) of the target protein. For example, the SAV W135A on the protein Q96E14 “Abolishes interaction with RMI1, TOP3A and BLM.“, and we thus assigned the label −1 at the molecular phenotype “interaction” class. We assigned the label 0 when the mutation does not alter the ability of the protein to interact with others, such as the K100A SAV on Q96E14, which “Does not affect interaction with RMI1, TOP3A and BLM”. We assigned the label 1 to the (rarer) SAVs that increase the interaction affinity of the target protein or that confer the ability to interact with novel partners (e.g. K73M SAV on protein Q16644 causes “Higher affinity toward PCH2″). We considered the phenotype interaction-related only when another protein (or gene) is mentioned. In the case of altered interaction with smaller molecules we annotated it in the “Binding” class. In the interaction class we also considered self-interactions (e.g. dimerization, trimerization, homodimerization). An example of this is R77E on Q8WXF7, which “Abolishes homodimerization” and it has thus been annotated as −1. We considered the interaction with the cell membrane as an aspect better described by the subcellular localization class.

#### 2.2.2 Binding

We used this class to label the change of binding affinity of the target protein with small molecules and nucleic acids (but not proteins). We annotated every SAV-driven decrease in affinity as −1 (e.g. D176N on P09936 causes a “6-fold decrease in affinity for ubiquitin ethyl ester”) and every increase as 1 (e.g. T104A on protein Q03426 causes “Approximately 4-fold increase affinity for ATP. Normal affinity for mevalonate”). SAVs that explicitly do not cause change in the ability of the protein to bind small molecules are labeled as 0 (e.g. A431T on Q07869 causes “No effect on heterodimerization with RXRA nor on DNA binding.”)

#### 2.2.3 Function

This class describes the effect that each SAV has on the protein function, which indeed depends on each target protein. To annotate this field we thus manually checked what is the function carried out by each protein. We assigned the label −1 to SAVs that impairs function, such as C320S on P42575, which causes “Loss of function”, and R1114E on Q96QB1 which causes “No catalytic activity”. We also label as −1 SAVs that just reduce the protein function (e.g. D675A on Q9H0M0 “Reduces ubiquitin transfer”), which could be an enzymatic activity (e.g. K294A on Q8WTS6, which “Significantly reduces the catalytic activity” and D297A on Q9Y2G5, which “Reduces enzyme activity”). We used the label 0 for SAVs that do not affect the function (e.g. Q270E on Q9Y3R4 has “No effect on enzyme activity”) and the label 1 when the SAV induces an increase of the protein activity or function (e.g. E246M on Q8WVQ1 “Increases activity 5-fold”).

In some cases we considered the function of the protein in the broader sense, such as a part of a signaling patwhay (e.g. V61Q on P15260 causes “Loss of function in the interferon-gamma-mediated signaling pathway”) or in the case of the Golgi to ER traffic protein 4 homolog (Q7L5D6) on which the SAV D84K “Reduces tail-anchored protein delivery”. Both of these examples were annotated with −1 (function).

#### 2.2.4 Post-translational modifications (PTMs)

We also annotated the most common changes in the protein PTMs due to SAVs. We explicitly labeled the changes in phosphorylation, ubiquination, glycosylation, sumoylation, acetylation, methylation, ribosylation, and we used the “other ptm” class to group the rarer types of PTMs. It is important to notice that we listed in these classes the PTMs that are passively undergone by the mutated protein, ensuring that the SAV disrupts the PTM on the mutated (target) protein (e.g. T432A on P68104 “Abolishes phosphorylation by PASK”, where T432 is a phosphothreonine on the mutated protein). On the contrary, when the phosphorylation *is performed* by the target protein (e.g. a kinase), and the SAV impairs the protein's ability to phosphorylate or induce phosphorylation, the SAV is labeled as −1 in the “function” class, because the target protein function (i.e. to phosphorylate other proteins) is affected by the SAV. This distinction makes HPMPdb conceptually different from existing PTM databases, in which only the passive meaning of the PTM annotation is considered. An example of a phenotypic annotation mentioning a PTM change that we classified as a −1 label in the “function” class is K181E on Q02297, since it causes the protein to be “Defective in integrin-binding and in inducing ERBB3 phosphorylation”.

In some cases, a SAV alters both the ability of a protein to perform phosphorylation and the phosphorylation of the protein itself (e.g. K78I on Q15831, which causes ”Loss of kinase activity, leading to greatly reduced autophosphorylation”) and we thus labeled these SAVs with a −1 on both the “phosphorylation” and the “function” classes. Similarly to the previous class labels, we used −1 for SAVs disrupting a certain PTM, 1 for SAVs introducing a novel PTM and 0 when the SAVs do not alter the known PTMs.

#### 2.2.5 Subcellular localization

This class label describes the SAVs that cause a change in the expected subcellular localization of the target protein (e.g. Y149A on P47871 “Abolishes expression at cell surface and glucagon binding”), or its ability to move between different cellular compartments (i.e S446A on Q13568 “Abolished nuclear translocation”). In this case we used only the 1 and 0 labels. We labeled as 1 the SAVs that changed the wildtype localization or the protein movement (e.g. expression at the cell membrane), both by impeding an expected localization or introducing a novel one (e.g. K286A on P17028, which causes a “Partial cytoplasmic accumulation” on a protein that is expected to be found in the nucleus). We labeled as 0 the SAVs that do not alter the wildtype localization.

#### 2.2.6 Mimetic PTM

This class labels all the SAVs that are listed as mimetic for a certain PTM (e.g. phosphomimetic) or that cause the target protein to mimick the state due to a certain PTM (e.g. permanent activation or inhibition). We only used the label 1 to indicate the mimetic property of the SAV. Two examples are T222E on P49137, which “Mimicks phosphorylation state and constitutive protein kinase activity” and S209D on P06730, which is described as “Phosphomimetic mutant”.

#### 2.2.7 Folding

This class describes all the SAVs that alter some aspects of the protein related to its folding or its stability. We used −1 to indicate SAVs that disrupt folding (e.g. K167A on P13747 “Impairs folding”) or reduce stability (e.g. R247Q on Q13394, “Decreased protein stability”). We labeled as 0 the SAVs that do not alter these aspects of the target protein (e.g. D4258A on P98160, “Retains proper folding. Reduced calcium ion binding.“) and as 1 the SAVs that increase stability (e.g. L84R on Q86W24 causes “Increased thermal stability of the Pyrin domain.”).

#### 2.2.8 Expression

This class label groups all the SAVs that alter the expression of the mutated proteins, such as N74A on P16422, which causes “Changed glycosylation pattern. Complete loss of glycosylation and substantial decrease in protein expression”. This label must not be confused with the subcellular localization-related occurrences of “expression”, such as K211A on P41595, which “Impairs protein folding and stability. Strongly reduced cell surface expression”. We used the −1, 0 and 1 labels for SAVs respectively decreasing, leaving unchanged and increasing protein expression.

### 2.3 Managing the heterogeneity of the natural language description and addressing inconsistencies in the annotations

While manually curating the −1, 0 and 1 labels of each phenotypic class, in many cases we had to simplify the natural language description of the SAV molecular phenotypes. In other cases, we encountered unclear or inconsistent descriptions. Here we describe in detail how we addressed these situations.

#### 2.3.1 Inconsistent descriptions

In some cases, the phenotypic description reported by Uniprot was contradictory. This is the case of K66Q on P61244 “Kept nuclear localization. Loss of nuclear localization” and M86L on Q9BU89 “No effect on iron-binding. Loss of iron-binding”. Also the N115Q, N157Q and N295Q SAVs on P06865 have an inconsistent description, since they are annotated as “No change of the catalytic activity associated with the alpha-chain. No catalytic activity associated with the alpha-chain”. Another example of inconsistent molecular phenotype description is D96E on Q9GZU7, which is annotated as “No effect. Completely abolishes phosphatase activity”. In these cases, we did not assign any label to the SAVs, leaving the corresponding row empty.

When the descriptions were only partially inconsistent, we still considered them. An example of this is W290A on P06748, which is annotated as “Partial destabilization of the structure. Complete destabilization of the structure”. The two statements in the description are not in agreement, but both of them could be associated with the −1 label of the class “folding”, and we thus used this label.

#### 2.3.2 Semantic equivalence between annotations

The natural language description of the molecular phenotypes can provide a wide variety of nuances and subtleties. Moreover, they can describe the effect of each SAVs with arbitrary level of detail, depending on who provided the description and on the experimental settings adopted. We thus observed situations like the SAVs on O95760, which carry the following descriptions: “Decreases affinity for IL1RL1″, “7-fold decrease in affinity for IL1RL1″, “Almost abolishes binding to IL1RL1″, “8-fold decrease in affinity for IL1RL1″, “Decreases affinity for IL1RL1”. Although the level of detail of these description varies, in order to reduce them to ML-suitable labels, we had to simplify them, and we thus annotated all of them with the −1 label in the “interaction” class. Other examples of phenotypic descriptions that we considered semantically equivalent are reported in Suppl. Mat. Section S1.

#### 2.3.3 Dealing with multi-phenotypic effects on the same and on different classes

When the same SAV is annotated with multiple phenotypic effects on multiple classes, we independently annotated one label per class. This is the case of R78A on Q9NRX4, which causes “Decreased affinity for substrate” (binding, −1) and “reduced catalytic activity” (function, −1). Another example is S90E on O00327, which is a “Phosphomimetic mutant” (mimeticPTM, 1) “with no effect on DNA binding” (binding, 0) “or CLOCK-ARNTL/BMAL1 transcriptional activity” (function, 0).

When multiple multiple phenotypic effects involving the same class are described, we used the following rule to ensure consistency in the labels: if only one of the mentioned phenotypes is altered and the others are wildtype-like, we ignored the wildtype and we reported the altered one. This is the case of E55G on Q92963, which causes a “Loss of interaction with AFDN, but not with RLF and RALGDS”. We thus annotated E55G as “interaction” −1 due to its negative effect on AFDN interaction. When the description of the phenotypes belonging to the same class reports both a enhanced and a reduced effect (e.g. N187L on A8MW95 “Increases strongly homodimerization. Decreases interaction with ATG14″), we did not annotate any label, because we should have indicated both −1 and +1 labels at the same time. Leaving the corresponding label empty will avoid providing inconsistent labels to the ML models. A similar example is N132V on Q9UIL1, which “Causes tetramerization and loss of interaction with FEZ1”. Another example of a missing label due to multiple opposite phenotypic effects within the same class is S233F on Q9ULV1, which causes a “Slightly increased signaling activity in presence of NDP/norrin and reduced signaling in presence of WNT3A.“. We thus would have had to report a −1 and +1 label for the class “function”.

In the cases where the annotations simply states “No effect.” (e.g. S37A on Q9BXJ7), we added the label 0 to all the classes that have been investigated on that specific protein (interaction and subcellular localization in the case of Q9BXJ7). The intuition behind this is that only the phenotypic effects that have been investigated for the target protein should be annotated as 0, and the other classes should be considered missing values, because they were likely not investigated at all by the experiments.

### 2.4 Webserved design and implementation

The webserver is freely available at hpmp.esat.kuleuven.be. It is a Django webserver running on Nginx. The data is stored as SQL database and the entire framework runs on a RedHat Linux in a Podman container.

## 3 Results and discussion

### 3.1 A machine learning-ready database of protein molecular phenotypes associated to Single Amino acid Variants

The Human Protein Molecular Phenotype Database (HPMPdb) contains 8007 highly-curated SAVs mapped on 1766 protein structures (see Methods for more details). Each of these SAVs has been associated by Uniprot with a natural language description of the molecular phenotype they cause on the carrier protein, reported from at least one publication in which the SAV effects have been experimentally determined. These phenotypic descriptions are very heterogeneous, sometimes inconsistent and in general very hard to automatically parse to extract Machine Learning (ML)-actionable labels for the predictions of these molecular phenotypes.

To overcome this gap, enabling data scientist to apply ML and automated analysis tools on this wealth of underused literature-derived phenotypic data provided by Uniprot, we manually curated the translation of these natural language phenotypic annotations into a controlled set of 15 ​ML-ready class labels, which can indeed be directly used in data analysis and ML pipelines/libraries. These labels describe the molecular phenotypic effects of SAVs on the carrier protein interactions, binding, function, post-translational modifications (PTMs) (e.g. phosphorylation, ubiquination, glycosylation, sumoylation, acetilation, methilation, ribosylation and “other PTM”), subcellular localization, mimetic PTM, folding and expression. The details of the translation rules we strictly followed to ensure consistency in the labels are explained in the Methods section.

The goal that we aim to achieve with HPMPdb is indeed to kickstart the next generation of variant effect predictors aiming at modeling the protein molecular phenotype elicited by each SAV. The vast majority of the existing variant-effect predictors take as input a SAV, but just aim at the binary prediction of a generic Deleterious or Neutral individual-level phenotype, thus considering only a binary phenotypic outcome with perfect Mendelian behavior. On the contrary, HPMPdb provides Bioinformaticians and Data Scientists with ML-ready class labels describing the molecular phenotype caused by each SAV at the protein level, thus allowing the development of a new generation of molecular phenotype predictors.

This level of detail could allow the multi-class or multi-label classification of the effects of SAVs at a much greater level of detail with respect to the conventional Neutral/Deleterious binary prediction, and, most importantly, associates each SAV to the phenotypes that they are directly causing, ignoring the cascade of feedbacks that they could trigger downstream.

Having in mind that detailed molecular features are likely needed in order to be able to model the protein molecular phenotype caused by each SAV, we ensured that each SAV in HPMPdb is indeed mapped on an experimentally determined protein structure from PDB (Berman et al., 2000). This will allow researchers to include structural information in their tools, favoring the development of novel end-to-end ML pipelines that can natively take protein structures as inputs, as proposed in the recently published ThermoNet (Bian et al., 2020) and in PyUUL (Orlando et al., 2022).

### 3.2 A user-friendly interface for browsing mutations

**Fig. 1:**
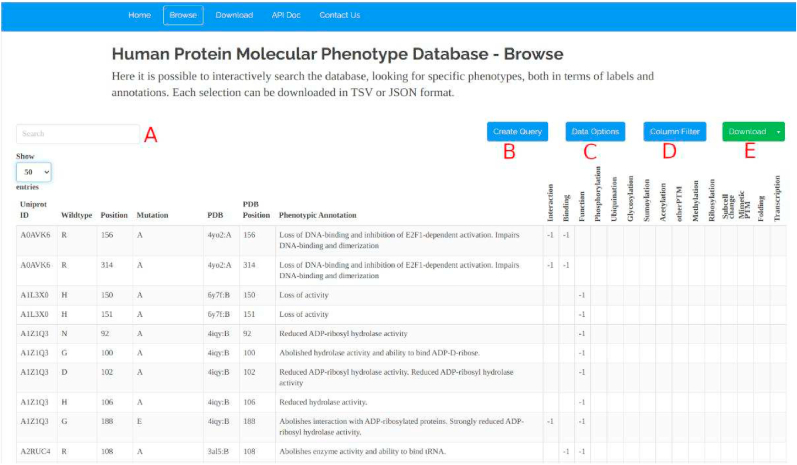
Figure showing the “Browse” page of HPMPdb database, where the user can interactively search for specific SAVs, proteins, keywords, class labels and use various filters to select subsets of the dataset (e.g. by collapsing columns).

The 8007 SAVs annotated on HPMPdb and the respective 15 classes can be downloaded directly in TSV, JSON or XML formats from our webserver, but in order to allow the user to easily perform pre-processing and transformation steps, we also provide an interactive visualization of the data in the “Browse” page (see Fig. 1).

This page contains an interactive table and functions that allow the user to interactively search for specific SAVs, proteins, keywords, class labels and use various filters to select subsets of the dataset (e.g. by collapsing columns or selecting specific columns). The results of each selection or search are interactively shown in the table. When the user is satisfied by the results, the visualized SAVs can be downloaded in TSV or JSON formats.

The fuzzy search field (see Fig. 1A) allows fast queries in the database, while the “Create Query” button (see Fig. 1B) allows the user to perform more specific ones, for example by searching a specific term within each of the columns.

The “Data Options” button (see Fig. 1C) allows the user to “Convert null values to zero” and/or “Convert values to absolute values”. In the first case, zeros are used to fill all the cells with missing values in the table. Missing values are used when a specific molecular phenotype is not specified in the SAV phenotypic description. In some ML settings, missing values could be difficult to deal with, and thus we allowed the user to automatically consider as “0 label” all of them. The “Convert values to absolute values” allows the user to transform all the −1 values (e.g. −1 in the interaction class indicates that a SAV disrupts an interaction with another protein that exist in the wildtype case) into “1” labels. This is helpful when the user is not interested in training a model to discriminate between gain (1) or loss (−1) of function SAVs, and just aims at the binary discrimination between SAVs that alter a specific molecular phenotype from SAVs that leave it at the wildtype levels.

The “Column Filter” button (see Fig. 1D) allows the user to select which columns should be shown, and thus be available for download. Moreover, it offers the ability to “Collapse PTM Columns”, thus pooling all the PTMs class labels (e.g. Phosphorylation, Ubiquination, etc.) into a unique “anyPTM” class, which is 1 when at least one of the PTM classes contains a 1. This operation is useful because some PTMs are observed only in few SAVs, and so there might thus be not enough examples to allow ML methods to properly learn to predict some of these classes.

The “Download” button (see Fig. 1E) allows the user to download the current selection showed in the table in JSON or TSV formats.

### 3.3 Database statistics

**Fig. 2:**
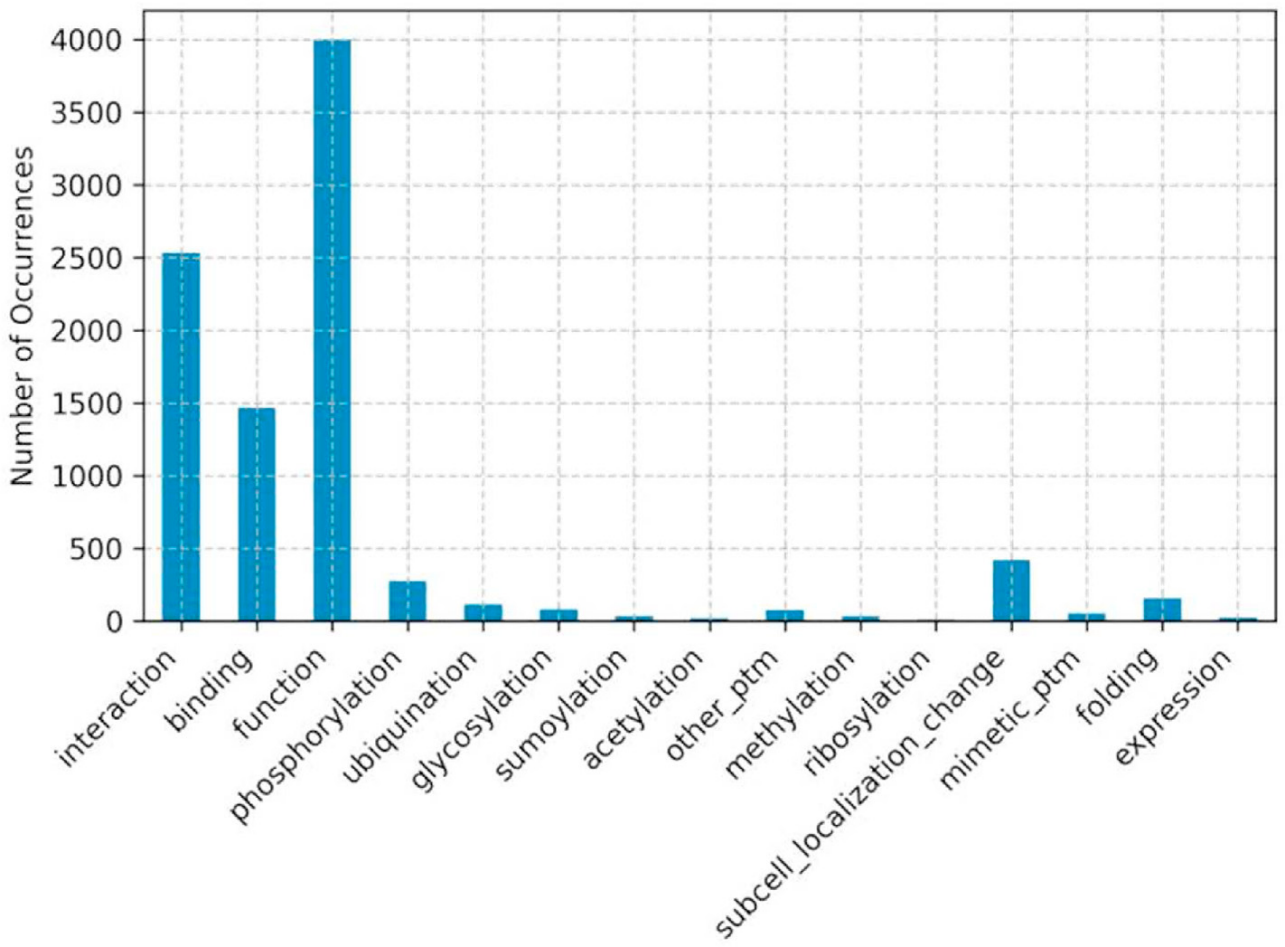
Figure showing the number of occurrences of each class label in the dataset.

HPMPdb contains 8007 SAVs mapped on 1766 proteins. For each SAV, we translated the phenotypic annotation provided by Uniprot into 15 ​ML-ready class labels (see Methods for more details). Fig. 2 shows the number of occurrences of each of these molecular phenotype classes among the dataset. The most represented class is the function, with 4000 labels indicating whether the SAV reduces/impairs it (label −1), leaves it unchanged (label 0) or causes a gain of function (label 1). The second most common is the interaction class, which similarly describes the effect of each SAV on the ability of the protein to interact with other proteins, indicating as −1 a reduced or impaired interaction as 0 a wildtype behavior and as 1 a gain of interaction due to the SAV. The third most represented class, with slightly less than 1500 instances is the binding, which describes the effect of the SAVs on the ability of the protein to bind small molecules or nucleic acids. The remaining classes are much sparser, with less than 500 instances each. To overcome this lack of labels, the Post Translational Modification (PTM) classes can be conveniently collapsed into a single class (anyPTM) from the interface on the webserver, pooling them into 961 “anyPTM” instances.

**Fig. 3:**
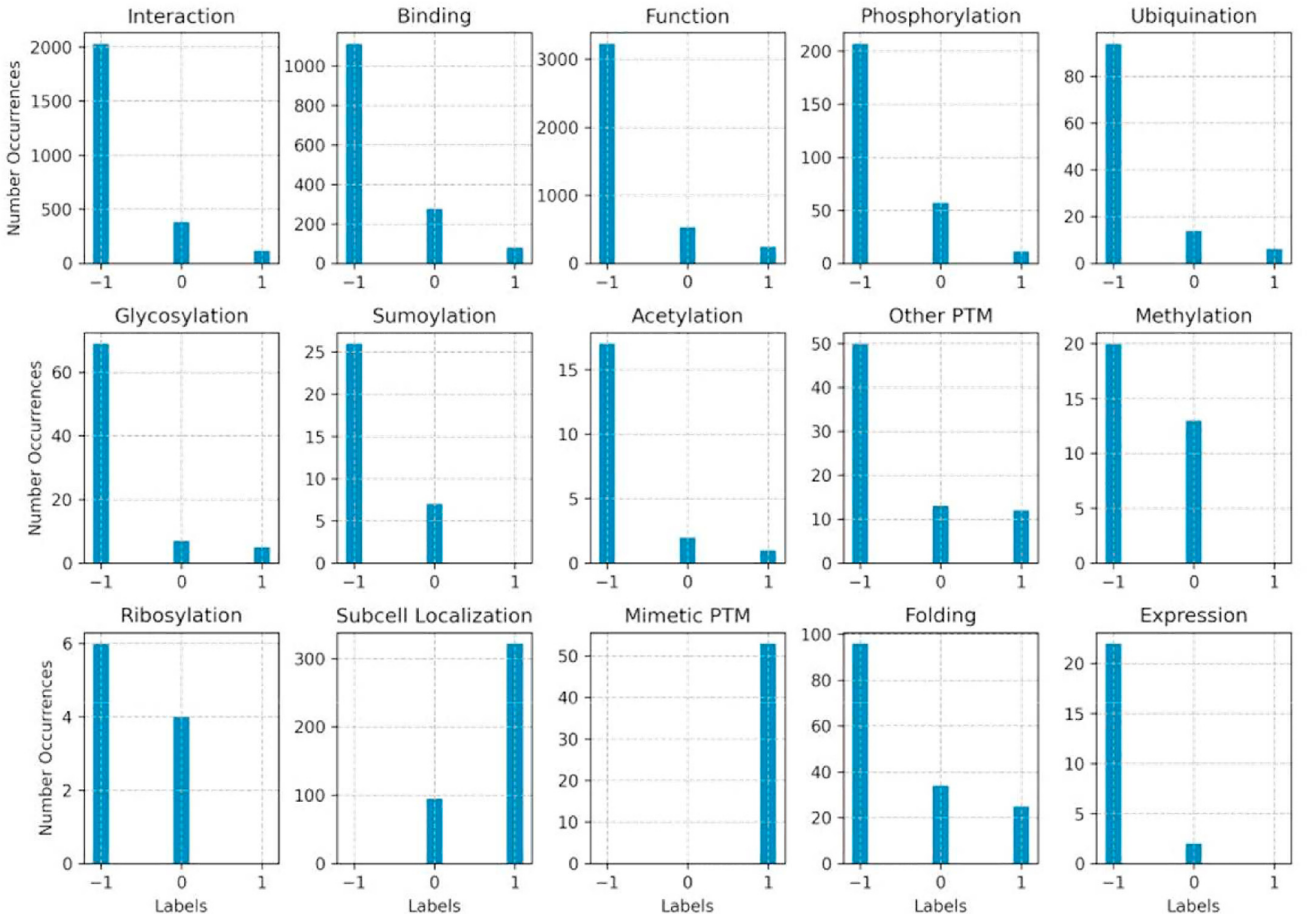
Figure showing, for each class label, the proportion of the SAVs annotate to reduce or eliminate the corresponding phenotype (label −1), to leave it unchanged (label 0) and to cause a gain of function (label 1).

Fig. 3 shows, for each class, the proportion of the −1, 0 and 1 labels, indicating how many SAVs have a detrimental, neutral or positive effect on the wildtype phenotype. We can see that most of the SAVs generally reduce the expected wildtype phenotype levels, and are thus assigned a −1 label. The exception in our annotations are the MimeticPTM and the Subcellular localization classes. As explained in Methods, the first describes SAVs that mimic a protein with a certain PTM (e.g. a phosphomimetic SAV that mimics a phosphorylated protein by for example activating or deactivating it permanently). In this case, we annotated as 1 the occur-rence of each of the mimetic PTM SAVs. For what concerns the Subcellular localization, we only distinguished between SAVs that do not alter the localization (0) and SAVs that change it (1) because it was not straightforward to determine a “positive” or “negative” change in localization, as it is for example for an increased or reduced enzymatic activity or binding affinity.

### 3.4 How to use HPMPdb to benchmark molecular phenotype predictors

Building a protein molecular phenotype predictor offers different practical and conceptual challenges with respect to building a conventional variant-effect predictors that aims at the “Deleterious/Neutral” binary discrimination. Since goal of the annotations in HPMPdb is to be used to train test and benchmark novel predictors, we describe here some recommended best practices and some possible approaches.

#### 3.4.1 Sequence-identity based stratified cross-validation

The protein sequences that we observe have a complex and intertwined evolutionary history, and a tight dependence between sequence, structure and function. For this reason, when training. testing and validating ML methods in the Bioinformatics context, it is crucial to stratify the train/test or the cross-validation folds in function of the protein sequence identity (SI), ensuring that proteins assigned to different train/test sets share a very low sequence similarity (Grimm et al., 2015). A SI threshold around 20% or 30% is generally considered acceptable. From the Download page of our webserver we thus provide the sets for a 5 and 10-folds cross-validation, stratiﬁed withBlastclust (Altschul et al., 1990) with a 20% SI threshold at 90% sequence coverage. We also provide the list ofthe Blastclust clusters, in case the users wanted to deﬁne their own SI-based validation sets.

#### 3.4.2 Choosing among the possible prediction tasks

While conventional variant-effect predictor perform a binary discrimination between deleterious and neutral variants, in the case of HPMPdb, the 15 class labels allow a much wider variety of prediction tasks to be solved.

The 15 HPMPdb class labels represent independent phenotypic annotations (e.g. a SAV might alter one or more of the 15 phenotypes). This means that the most straightforward approach would be the multi-task prediction settings, where a model is designed and trained to concurrently predict the effect of a SAV on multiple phenotypes. Although the multi-task prediction is not straightforward to achieve with some conventional ML methods (e.g. Random Forest, Support Vector Machines), it is standard practice for Neural Networks (NN) (Raimondi et al., 15AD), where the final layer of the NN has one output layer for each task (i.e. each phenotype class in the HPMPdb context).

Given this multi-task approach, few conceptual choices remain to be made in order to buld a ML model suitable for the HPMPdb data. For example, the user should decide whether for each phenotypic class (task) they wish to predict the “Decreased”, “Wildtype” or “Increased” phenotypic effect of the SAV (corresponding respectively to the −1, 0 and 1 labels), or just discriminate between SAVs that “Alter the phenotype” (labels −1, 1) versus “Wildtype” SAVs (label 0). The first option would make the multi-task prediction problem a series of concur-rent 3-classes multi-class predictions, while the second would lead to a conventional multi-task problem where each of the concurrent prediction is a binary classification.

Moreover, the users should determine the path to follow depending on the label availability for each class, in relation with the characteristics of the chosen ML model. For example, from what emerges from Fig. 3, no positive (1s) labels are available for the Methylation, Expression and Sumoylation classes, meaning that all the SAVs affecting them just have an impairing or wildtype effect. Also the Subcellular localization class has been annotated only with 0 and 1 labels, indicating respectively a wildtype localization or a change in the protein localization. A similar reasoning goes for the MimeticPTM class. This means that the −1, 0 and 1 labels prediction is not possible for all the 15 molecular phenotype classes in HPMPdb. In general, from Fig. 3 it emerges that the SAVs associated to the 1 label (e.g. gain of function) are rarer than the −1 label, and thus it might be reasonable to conclude that there are not enough data to frame the ML problem as a multi-task prediction of −1, 0 and 1 outputs, and that the binary discrimination between “Alters the phenotype” (labels −1, 1) versus “Wildtype” (label 0) option would be more realistic in terms of data availability. This can be achieved by just clicking on “Data Options” and “Convert values to absolute values” on the Browse page of our webserver (see Fig. 1).

#### 3.4.3 The role of missing phenotypic annotations

The class labels we provide in HPMPdb (see the table in Fig. 1) are extremely sparse, because for each SAV the phenotypic annotations referred to one or at most few of the 15 phenotypic classes we defined. This is due to the fact that for each SAV and proteins, only specific phenotypes have been experimentally assessed.

In order to maintain consistency, we decided to leave empty the class label associated to unspecified phenotypes, because we could not assert anything about them. Nevertheless, on our webserver we allow the users to automatically convert the missing values into 0 labels by just clicking on “Data Options” and “Convert null values to zero” on the Browse page of our webserver (see Fig. 1). Even though this action carries the strong assumption that unobserved phenotypes are wildtype level, while it is not likely that all those phenotypes have been experimentally investigated, we leave this decision to the users.

#### 3.4.4 Collapsing phenotypes into more generic classes

From Figs. 2 and 3 it clearly emerges that while thousands of labels are available for the interaction, binding and function classes, less than 500 labels are available for each of the other molecular phenotype classes, including the PTMs. Limited amount of available labels might impair the ML models generalization and increase the risk of overfitting. To partially solve this problem, we offer the option of collapsing all the specific PTMs into a single class (anyPTM) by clicking on “Column Filters” and “Activate Collapse”. The new anyPTM class that substitutes the 8 PTM classes now contains 1 when the SAV alters any PTM, 0 when all PTMs preserve the wildtype status and empty when no PTM annotation is available. This grouping allows the user to aggregate PTM labels into a less specific, but more populated, class, thus possibly simplifying the prediction task.

#### 3.4.5 Choosing suitable machine learning features to describe SAVs

Conventional variant-effect predictors use various types of features to contextualize both the effect of the target SAV on the carrier protein and the relevance of that protein for the welfare of the organism (Raimondi et al., 2016, 2017; Calabrese et al., 2009). It turns out that, depending on the dataset, the protein-related features (e.g. protein domain composition, gene relevance, involvement in pathways) carry a very strong signal for the prediction of the Deleteriousness of a SAV (Raimondi et al., 2016, 2017). This is due to the fact that the biological relevance of a protein is a *sine qua non* condition for a SAV to cause a phenotypic effect that can be detected at the individual level. For example, in (Raimondi et al., 2016) it emerged that all of the 500 SNVs in the 2011 version of Humsavar (Apweiler et al., 2004) mapped on the Olfactory Signaling Pathway were Neutral SAVs, regardless of their impact on the protein, and that in general the membership of a protein to certain pathways is a strong predictor of the Neutral or Deleterious status of any SAV mapped on it (Raimondi et al., 2016). A similar behavior has also been detected in cancer driver variant predictors, showing that a detailed identification of the mutated genes carries a sufficiently strong predictive signal regardless of the impact of the SAV itself (Raimondi et al., 2021).

This gene-centric signal is not likely to be so crucial when training and testing a ML model on HPMPdb, because the SAVs' labels directly refer to the molecular phenotype that they cause on each protein, and thus features that describe this molecular-level effect should be prioritized instead. The classical Multiple Sequence Alignments (MSAs)-derived conservation indices (Raimondi et al., 2016; Ng and Henikoff, 2001; Calabrese et al., 2009) will likely help, but, taken alone, they are at risk of not being specific enough to distinguish between different phenotypic classes. For example a SAV altering an interaction hotspot and one disrupting folding are both likely to hit a very conserved position in the MSA, but the conservation alone is not likely to be sufficient to discriminate between them. We thus hypothesize that a combination of features describing various SAV-related molecular aspects might be necessary to achieve reasonable prediction performance on HPMPdb. Following this last example, adding as a feature the Relative Solvent Accessibility (RSA) of the mutated region might help the discrimination, because interaction hotspots are likely to be conserved positions located on the protein surface, while folding-crucial positions are conserved positions more likely to be found in the protein hydrophobic core.

#### 3.4.6 The role of structure-based features

Collecting enough detailed features to contextualize the all the fine-grained molecular-level aspects necessary to correctly predict each phenotype class while relying only on sequence-derived information (e.g. sequence-based RSA predictors, protein stability predictors, DNA binding predictors, etc.) might be difficult, because some of these tools might not be able to provide reliable enough predictions or annotations. For this reason, in the data collection phase we used SIFTS (Velankar et al., 2012) to ensure that every protein in HPMPdb had at least a partial correspondence with a PDB structure (Berman et al., 2000), and we provide the sequence-to-structure mapping provided by SIFTs in our main dataset table (see Fig. 1). In this way, we encourage the users to use the 3D protein structure data available on PDB to contextualize further the possible molecular effects of each SAV, either by extracting features from the corresponding protein structure or by building fully end-to-end ML approaches that take the PDB structure (or a portion centered on the target SAV) and directly use the 3D volume and its content as input for a ML pipeline (Orlando et al., 2022) (e.g. a Neural Network). A similar approach has been adopted to predict protein thermal stability (Bian et al., 2020) and in the PyUUL library (Orlando et al., 2022).

## 4 Conclusion

In this paper we present the Human Protein Molecular Phenotype database (HPMPdb), which is freely available at hpmp.esat.kuleuven.be. It contains 8007 Single Amino acid Variants (SAVs) mapped on 1766 human proteins. For each SAV we manually curated the translation of the natural language description of its molecular phenotype into 15 Machine Learning (ML)-ready class labels, thus providing a dataset that Bioinformaticians and Data Scientists can easily use to train, test and benchmark the first generation of variant-effect predictors devoted to the prediction of detailed protein molecular phenotypes instead than the conventional Deleterious/Neutral effect for the carrier individual. To the best of our knowledge, HPMPdb is the first resource that carries this kind of protein-centric molecular phenotype data, and thus it could be instrumental for the development of a new generation of more detailed variant-effect predictors.

## Funding

DR is funded by an FWO post-doctoral fellowship. The Switch laboratory is supported by the 10.13039/501100004727Flanders Institute for Biotechnology (VIB, grant no. C0401); 10.13039/501100004497KU Leuven (postdoctoral mandate PDM/19/157 to G.O.); and 10.13039/501100003130The Research Foundation – Flanders (FWO, project grants G053420N and SBO S000722N).

## CRediT authorship contribution statement

**Daniele Raimondi:** Conceptualization, Methodology, Data curation, Writing – review & editing. **Francesco Codicè:** Data curation, Methodology, Visualization, Software. **Gabriele Orlando:** Conceptualization, Methodology, Data curation, Writing – review & editing. **Joost Schymkowitz:** Writing – review & editing, Funding acquisition. **Frederic Rousseau:** Writing – review & editing, Funding acquisition. **Yves Moreau:** Writing – review & editing, Funding acquisition.

## Declaration of competing interest

The authors declare that they have no known competing financial interests or personal relationships that could have appeared to influence the work reported in this paper.
