# Supplementary material for: HPMPdb: A machine learning-ready database of protein molecular phenotypes associated to human missense variants
Source: Curr Res Struct Biol. 2022 May 13;4:167–74. doi: 10.1016/j.crstbi.2022.04.004 (PMC9166469; doi:10.1016/j.crstbi.2022.04.004)
Supplement: Multimedia component 1 [file mmc1.pdf]

Supplementary Material

S1 Additional examples of semantically equivalent phenotypic annotations

P27338 C 5 S 6yt2:A 5 No loss of activity.
P27338 C 156 S 6yt2:A 156 Complete loss of activity.
P27338 T 158 A 6yt2:A 158 Dramatic loss of activity.
P27338 C 172 S 6yt2:A 172 No loss of activity.
P27338 C 192 S 6yt2:A 192 No loss of activity.
P27338 I 199 F 6yt2:A 199 Alters specificity towards synthetic inhibitors.
P27338 C 297 S 6yt2:A 297 No loss of activity.
P27338 C 312 S 6yt2:A 312 No loss of activity.
P27338 C 365 S 6yt2:A 365 Complete loss of activity.
P27338 H 382 R 6yt2:A 382 Significant loss of activity.
P27338 K 386 M 6yt2:A 386 No loss of activity.
P27338 C 389 A 6yt2:A 389 Complete loss of activity.
P27338 C 389 S 6yt2:A 389 No loss of activity.
P27338 S 394 A 6yt2:A 394 No loss of activity.
P27338 C 397 S 6yt2:A 397 Complete loss of activity.

Another example is the following:

Q13093 S 273 A 3f98:A 273 Loss of activity.
Q13093 D 286 A 3f98:A 286 Almost no activity.
Q13093 D 286 N 3f98:A 286 Diminishes activity.
Q13093 D 296 A 3f98:A 296 Loss of activity.
Q13093 D 296 N 3f98:A 296 Loss of activity.
Q13093 D 304 A 3f98:A 304 No change in activity.
Q13093 D 338 A 3f98:A 338 Activity is higher than wild-type.
Q13093 H 351 A 3f98:A 351 Loss of activity.

Another example is the following:

P41180 R 69 E 5fbk:A 69 Abolishes G-protein coupled receptor signaling pathway.
P41180 N 102 I 5fbk:A 102 Abolishes G-protein coupled receptor activity.
P41180 T 145 A 5k5t:A 145 Abolishes G-protein coupled receptor activity.
P41180 S 147 A 5k5t:A 147 Nearly abolished G-protein coupled receptor activity.
P41180 S 170 A 5k5t:A 170 Abolishes G-protein coupled receptor activity.
P41180 Y 218 S 5k5t:A 218 Abolishes G-protein coupled receptor activity.
P41180 E 297 I 5k5t:A 297 Abolishes ability to sense calcium or magnesium levels.
P41180 S 417 L 5k5t:A 417 Abolishes G-protein coupled receptor signaling pathway.
P41180 W 458 A 5k5t:A 458 Decreased G-protein coupled receptor signaling pathway.
